# Isolation of a human SARS-CoV-2 neutralizing antibody from a synthetic phage library and its conversion to fluorescent biosensors

**DOI:** 10.1038/s41598-022-19699-z

**Published:** 2022-09-15

**Authors:** Haimei Li, Bo Zhu, Baowei Li, Limei Chen, Xuerao Ning, Hang Dong, Jingru Liang, Xueying Yang, Jinhua Dong, Hiroshi Ueda

**Affiliations:** 1 Weifang Key Laboratory for Antibodies Medicine, School of Life Science and Technology, Weifang Medical University, Weifang, China; 2 Laboratory for Chemistry and Life Science, Institute of Innovative Research, Tokyo Institute of Technology, Yokohama, Japan; 3 School of Basic Medical Sciences, Peking University, Beijing, China; 4 World Research Hub Initiative (WRHI), Institute of Innovative Research, Tokyo Institute of Technology, Yokohama, Japan; 5 Present Address: School of Rehabilitation Sciences and Engineering, University of Health and Rehabilitation Sciences, Qingdao, China

**Keywords:** Antibody isolation and purification, Infection, Biochemical assays

## Abstract

Since late 2019, the outbreak of severe acute respiratory syndrome coronavirus 2 (SARS-CoV-2) and the resultant spread of COVID-19 have given rise to a worldwide health crisis that is posing great challenges to public health and clinical treatment, in addition to serving as a formidable threat to the global economy. To obtain an effective tool to prevent and diagnose viral infections, we attempted to obtain human antibody fragments that can effectively neutralize viral infection and be utilized for rapid virus detection. To this end, several human monoclonal antibodies were isolated by bio-panning a phage-displayed human antibody library, Tomlinson I. The selected clones were demonstrated to bind to the S1 domain of the spike glycoprotein of SARS-CoV-2. Moreover, clone A7 in Fab and IgG formats were found to effectively neutralize the binding of S protein to angiotensin-converting enzyme 2 in the low nM range. In addition, this clone was successfully converted to quench-based fluorescent immunosensors (Quenchbodies) that allowed antigen detection within a few minutes, with the help of a handy fluorometer.

## Introduction

The worldwide outbreak of severe acute respiratory syndrome coronavirus 2 (SARS-CoV-2) started in late 2019 and is still ongoing. The rapid worldwide spread of coronavirus disease 2019 (COVID-19) has resulted in a global health crisis that poses great challenges to public health and clinical treatment, in addition to threatening human health and the global economy^1^.

The SARS-CoV-2 virus has a diameter of 75–160 nm, and its genome is a continuous linear single-stranded RNA that successively encodes nuclear proteins, envelope proteins, membrane proteins, and spike proteins, also known as S-proteins or S^1^. The S protein is arguably the most important protein on the viral surface. Its main function is to determine the host range and specificity of the virus and to integrate it with the host cell membrane receptor, to achieve cell infection. Spike protein has two subunits, S1 and S2. The receptor-binding domain (RBD) in S1 interacts strongly with human angiotensin-converting enzyme 2 (ACE2), while S2 contains the basic elements required in the membrane fusion process, to achieve the fusion of viruses and cells^2,3^. Zhou et al. found that SARS-CoV-2 can bind to the ACE2 receptor in humans, bats, civet cats, and pigs, which makes ACE2 inevitable for viral infection. The group then further analyzed the complex structure of the complete ACE2 protein and the RBD of the S protein using cryo-electron microscopy, while utilizing the previously analyzed ACE2 protein structure^4^. Wang and Zhang clarified the crystal structure of the complex of RBD and ACE2 using X-ray diffraction and found the interaction site between ACE2 protein and the viral S protein^5^. The affinity between SARS-CoV-2 and ACE2 was found to be 10–20-fold higher than that between SARS-CoV-1 and ACE2, which is considered to be the main reason for the super-infectivity of SARS-CoV-2^2,6^.

Various treatments for COVID-19 have been proposed in many countries^7^. At present, there are two main therapeutic strategies for COVID-19. The first is the use of traditional drugs, such as antiviral drugs and immunomodulators, including small molecule drugs, remdesivir, chloroquine, and hydroxychloroquine, combined use of the two drugs, lopinavir and ritonavir, and the three drugs, lopinavir, ritonavir, and interferon^8,9^. Unfortunately, there is no obvious therapeutic effect and some serious side effects^1^. The second is a biological therapeutic method related to the inhibition of viral infection in host cells, including SARS-CoV-2 S protein-neutralizing antibody, recombinant human soluble ACE2 protein, and new biological products designed against the key checkpoints in the life cycle of SARS-CoV-2^8–10^. Antibody drugs play an important role in the treatment of infectious diseases, autoimmune diseases, and tumors. In the early stage of the pandemic, some researchers encouraged the use of serum from convalescent patients to treat severe patients, which showed a degree of effect, indicating that SARS-CoV-2 antibody can effectively weaken the infective ability of the virus. S protein-neutralizing antibodies can neutralize virus toxicity by blocking the binding of ACE2 to S protein^2,11^. Therefore, the S monoclonal antibody can block the binding of virus to ACE2-positive cells and reduce the severity of viral infection. Single domain antibodies or nanobodies can neutralize spike proteins by targeting the hidden epitopes at the interface of trimer spike proteins, thus blocking the binding to ACE2 upon recognition by RBD with different conformations^12–14^. Barnes et al. analyzed and classified the structural changes of antibodies with different neutralizing activities in the process of binding with ACE2 and S-RBD domains^15^. At present, cocktail therapy with monoclonal antibodies against all coronavirus strains is also being administered, as it expands the neutralization potential, thus providing a therapeutic scheme for dealing with the mutation of virus strains^10,16–19^. So far, the U.S. Food and Drug Administration has approved human antibodies such as Regen-COV (Casirivimab and Imdevimab), Bamlanivimab/Etesevimab, Sotrovimab, Actemra (Tocilizumab), Evusheld (tixagevimab co-packaged with cilgavimab), and Bebtelovimab for treatment of mild to moderate COVID-19 (https://www.fda.gov/emergency-preparedness-and-response/mcm-legal-regulatory-and-policy-framework/emergency-use-authorization#coviddrugs).

With the rapid spread of SARS-CoV-2, there is a need for effective detection technology that can support rapid monitoring and disease management, and thus, help control the pandemic. Nucleic acid detection using reverse transcription-polymerase chain reaction is the main method for the diagnosis of SARS-CoV-2 infection, but it is time- and labor-consuming. Immunochromatography (lateral flow assay, LFA) can directly detect viral proteins with colloidal gold-labeled antibodies, and is now widely used as an antigen-test. However, the accuracy of detection in early infection before the symptom onset is not always good and it results in false negatives^24^. Also, the method is semi-quantitative and involves a detection period of more than 10 min. Therefore, there is a requirement for faster and more quantitative detection methods.

Quenchbody (Q-body) is an antibody fragment that is site-specifically labeled with fluorescent dye(s) near the antigen-binding site^20^. When the antigen is absent, the fluorescent dye moves to the inside of the antibody, and its fluorescence is quenched by tryptophan residues. When the Q-body binds to its antigen, the conformation of the antibody changes, and the fluorescence intensity of the fluorescent dye is restored. The antigen can be quantified by measuring the change in the fluorescence intensity of the Q-body. The operation of the Q-body assay is simple, because only the fluorescence-quenched body immunosensor is mixed with the sample to be tested, and the determination time is tens of seconds to a few minutes^21,22^. To date, Q-body technology has been developed for the detection of small molecules^23,24^, peptides^25^, and proteins or their phosphorylation^26–29^, and a variety of Q-body preparation methods have been developed^30–32^.

In this work, we tried to obtain human antibody fragments that bind to the SARS-CoV-2 S protein to make the antibodies that work both in therapy and rapid sensing. Based on the pioneering works of George Smith^33^ and Greg Winter^34^ the two variable region fragments of the H and L chains of an antibody responsible for antigen-binding could be connected with the help of a short linker (single-chain Fv, scFv) and displayed on phage. Since this technology established a direct connection between genotype and phenotype, it made the screening of antigen binders simple and efficient. In this study, an antibody capable of binding to the S protein of SARS-CoV-2 was selected from the Tomlinson I phage-displayed synthetic scFv library^35^. It was shown that an IgG derived from the binder clone successfully neutralized the binding of a pseudovirus to ACE2-overexpressing cells. A specific clone was used to prepare Q-bodies for the rapid detection of viral proteins and pseudovirus particles.

## Results

### Selection of spike-specific monoclonal antibodies from the human synthetic phage display antibody library

**Figure 1:**
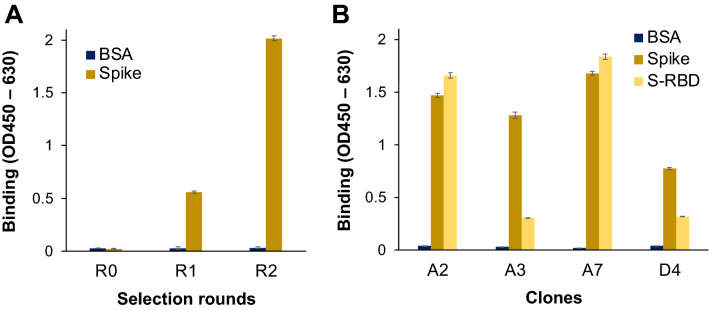
Polyclonal phage ELISA to confirm enrichment of the SARS-CoV-2 spike protein-binding phage antibody during bio-panning (**A**) and monoclonal phage ELISA results to identify the antigen-binding activity and specificity of individual antibodies (**B**). R0, R1, and R2 denote the original library and libraries obtained after rounds 1 and 2 of bio-panning, respectively. n = 3; Data have been expressed as mean ± standard deviation.

The Tomlinson I phage-displayed scFv library (7.3 × 10^12^ cfu/mL) was prepared in 2 mL sterilized PBS. Two bio-pannings of this library were performed on the immobilized SARS-CoV-2 S1 protein, to yield the sub-libraries R1 and R2, with titers of 5.2 × 10^12^ cfu/mL and 2.1 × 10^13^ cfu/mL, respectively. Enzyme-linked immunosorbent assay (ELISA) was performed to compare the antigen-binding ability of the polyclonal phages. As shown in Fig. 1A, when the binding affinities of the polyclonal phages R0, R1, and R2 to S1 protein were compared, the binding of phage solution R2 to S1 protein increased significantly, while the binding to bovine serum albumin (BSA) was weak and unchanged, as compared to that of the R0 phage, indicating that displayed antibodies against S1 protein were enriched during the bio-panning.

Cloning and testing of the R2 phages using ELISA led to the selection of four positive clones, A2, A3, A7, and D4. As shown in Fig. 1B, all the clones bound specifically to SARS-CoV-2 S1 protein, and among them, A2 and A7 bound to S-RBD with signals of similar intensity, suggesting that mainly A2 and A7 recognize the RBD of the S protein, while A3 and D4 may bind to other domains. By comparing the sequences of these genes with GenBank sequences, no identical sequence was found. The clone A7 that showed the strongest S-RBD binding activity was further investigated. The V_H_ sequence of A7 encoded 117 amino acids, and the CDRH2 and H3 including diversified residues (underlined) were identified as AIASSGYYTSYADSVKG and DTDTFDY, respectively. Also, the V_L_ sequence encoded 114 amino acids, and the CDRL2 and L3 were identified as AASTLQS and QQANSSPST, respectively, according to Kabat numbering scheme identified by abYsis (http://www.abysis.org/abysis/)^36^.

### Expression, purification, and affinity analysis of Fab fragments

**Figure 2:**
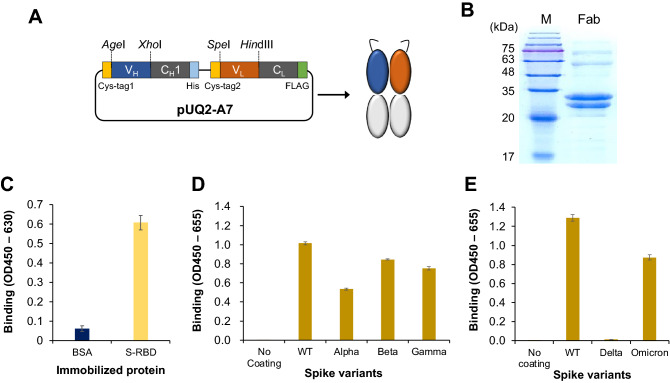
Construction of A7 Fab expression plasmid (**A**) and SDS-PAGE of the purified A7 Fab fragment (**B**). Cys-tag1, Cys-tag2: peptides containing cysteine residues for dye-labeling; M: Color Mixed Protein Marker (11–180 kDa; Solarbio). Original gel image is presented in Supplementary Fig. S3. The binding activity of A7 Fab to SARS-CoV-2 RBD (**C**), the full-length spike proteins isolated from Wuhan (WT) and the variants (**D-E**) were investigated by ELISA (n = 3). Data have been expressed as mean ± standard deviation.

The V_H_ and V_L_ genes of the obtained clones were transferred to the pUQ2 vector^21^, to express the N-terminal Cys-tagged human antigen-binding (Fab) fragment, as shown in Fig. 2A. The Fabs were expressed in *E. coli*, purified with Ni–NTA beads, and analyzed using sodium dodecyl sulfate–polyacrylamide gel electrophoresis (SDS-PAGE). As shown in Fig. 2B, the presence of two protein bands at approximately 28 kDa was observed for the purified A7 Fab fragment, probably representing the antibody Fd (= V_H_-C_H1_) and the light chain of the Fab, thus showing its successful expression and purification.

The antigen-binding affinity and specificity of A7 Fab were tested using ELISA (Fig. 2C). The A7 Fab bound to S-RBD with an absorbance of 0.62, which was significantly higher than that of BSA, which had an absorbance of 0.05, demonstrating that the A7 Fab antibody fragment can specifically bind to SARS-CoV-2 S-RBD. The ability to recognize five SARS-CoV-2 S protein variants by A7 Fab was also investigated (Fig. 2D,E). The spike protein of the variants of concern (VOC) Alpha, Beta, Gamma and Omicron can be recognized by A7 Fab with reasonable strength (> 50% signal) compared with the wild-type spike protein.

**Figure 3:**
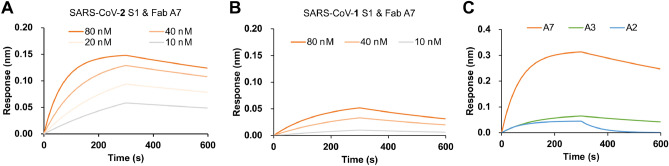
Kinetics analysis of A7 Fab and other variants using bio-layer interferometry, to evaluate their affinity to spike proteins of SARS-CoV-2 and SARS-CoV-1. (**A**) Sensorgram of A7 Fab against SARS-CoV-2 S1 protein. (**B**) Sensorgram of A7 Fab against SARS-CoV-1 S1 protein. (**C**) Sensorgram of different Fab variants against SARS-CoV-2 S1 protein.

The binding affinity and specificity of A7 Fab were further analyzed using bio-layer interferometry (BLI; Fig. 3A and Supplementary Table S1). The equilibrium dissociation constant *K*_D_ for SARS-CoV-2 S1 was calculated as 2.89 nM (R^2^ = 0.960). A similar measurement was also performed for S1 proteins derived from SARS-CoV-1 (isolate: WH20) (Fig. 3B). However, the response to SARS-CoV-1 S1 was much lower than that to SARS-CoV-2 S1. It was also found that the binding of Fab A7 to SARS-CoV-2 S1 (*K*_D_ = 2.9 nM) was stronger than that to RBD alone (*K*_D_ = 7.4 nM). These results indicated that the A7 Fab recognizes SARS-CoV-2 specifically. The responses of A2 Fab and A3 Fab were also compared with that of A7 Fab against SARS-CoV-2 S1 using BLI and found to be significantly lower than that of A7 Fab at 200 nM (Fig. 3C).

### The A7 Fab antibody fragment blocks ACE2 binding to S1 protein

**Figure 4:**
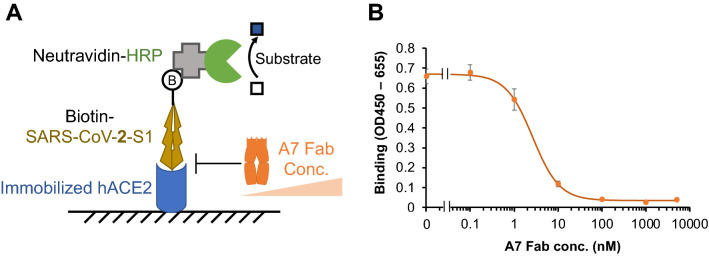
A7 Fab inhibits the binding of SARS-CoV-2 S1 protein to human ACE2. (**A**) The scheme for carrying out ELISA to verify the inhibition effect of A7 Fab. hACE2: recombinant human ACE2. (**B**) ELISA result showing that the binding between SARS-CoV-2 S1 and hACE2 is inhibited by A7 Fab. n = 3; Data have been expressed as mean ± standard deviation.

To investigate the possibility of infection-neutralization activity of A7, competitive binding of A7 Fab and S1 protein to immobilized ACE2 was carried out (Fig. 4A). As shown in Fig. 4B, at low concentrations of A7 Fab, biotinylated S1 protein bound to immobilized ACE2 and a strong signal was detected with Neutravidin-horseradish peroxidase (HRP) and HRP substrate. As expected, with an increase in A7 Fab concentration, the absorbance of the samples decreased at 450 nm. The binding of the S1 protein to ACE2 decreased to 5% after the addition of more than 100 nM of A7 Fab, proving that the A7 antibody can effectively block the binding of the S1 protein to ACE2. The 50% inhibition concentration IC_50_ of A7 Fab was estimated as 2.7 nM under the current assay conditions (with 10 nM S1 protein). To calculate the inhibition constant *K*_i_ of the A7 Fab, the affinity between ACE2 and S1 protein was measured (*K*_D_ = 31.8 nM). The *K*_i_ of A7 Fab against the ACE2 and S1 interaction was estimated as 2.1 nM.

### Preparation of A7 IgG and neutralization of viral infection

**Figure 5:**
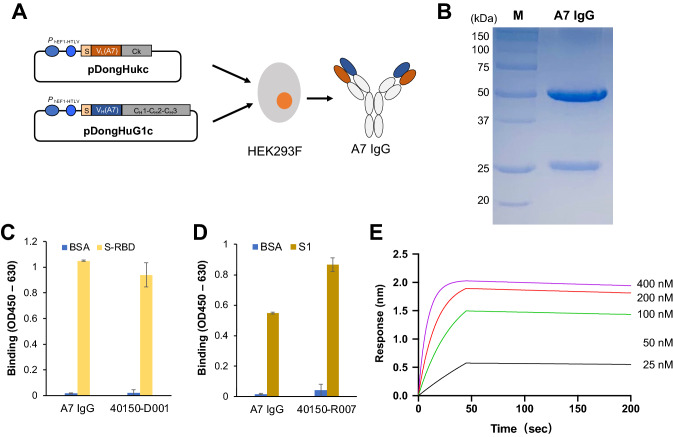
Expression and characterization of A7 IgG. (**A**) The scheme for expression of A7 IgG in the mammalian cells, HEK293F; (**B**) SDS-PAGE analysis of purified A7 IgG; (**C**,**D**) Antigen-binding ELISA of A7 and positive control IgGs; and (**E**) BLI sensorgram of A7 IgG. In (**C**,**D**), each protein (2 µg/mL) coated on the microplate well was reacted with 5 µg/mL A7 IgG. n = 3; Data have been expressed as mean ± standard deviation. Original gel image is presented in Supplementary Fig. S3.

To investigate the neutralization activity of this clone in more detail, we decided to prepare the full-length antibody and examined its effect to prevent SARS-CoV-2 S-pseudovirus infection. We used A7 variable region fragments to construct the vectors pDongHuG1c-A7 and pDongHukc-A7 for expression of full-length IgG. The two vectors were used to co-transfect HEK293F cells, following which the A7 IgG secreted from the cells was purified, as shown in Fig. 5A. Upon carrying out SDS-PAGE analysis (Fig. 5B) of the obtained protein, two clear bands were observed: one at 50 kDa, which is the heavy chain of the full-length antibody, and another near 25 kDa, which is the light chain of the antibody. Following that, ELISA was performed to verify antigen-binding activity of the IgG. As shown in Fig. 5, C and D, A7 IgG bound to S-RBD and S proteins, whose specificity was consistent with those of the phage-displayed scFv and Fab of A7. BLI was carried out to analyze the kinetic constant of A7 IgG to RBD, the sensorgram for which (shown in Fig. 5E) was then used to calculate the *K*_D_ value of A7 IgG as 9.37 × 10^–10^ M after global fitting.

**Figure 6:**
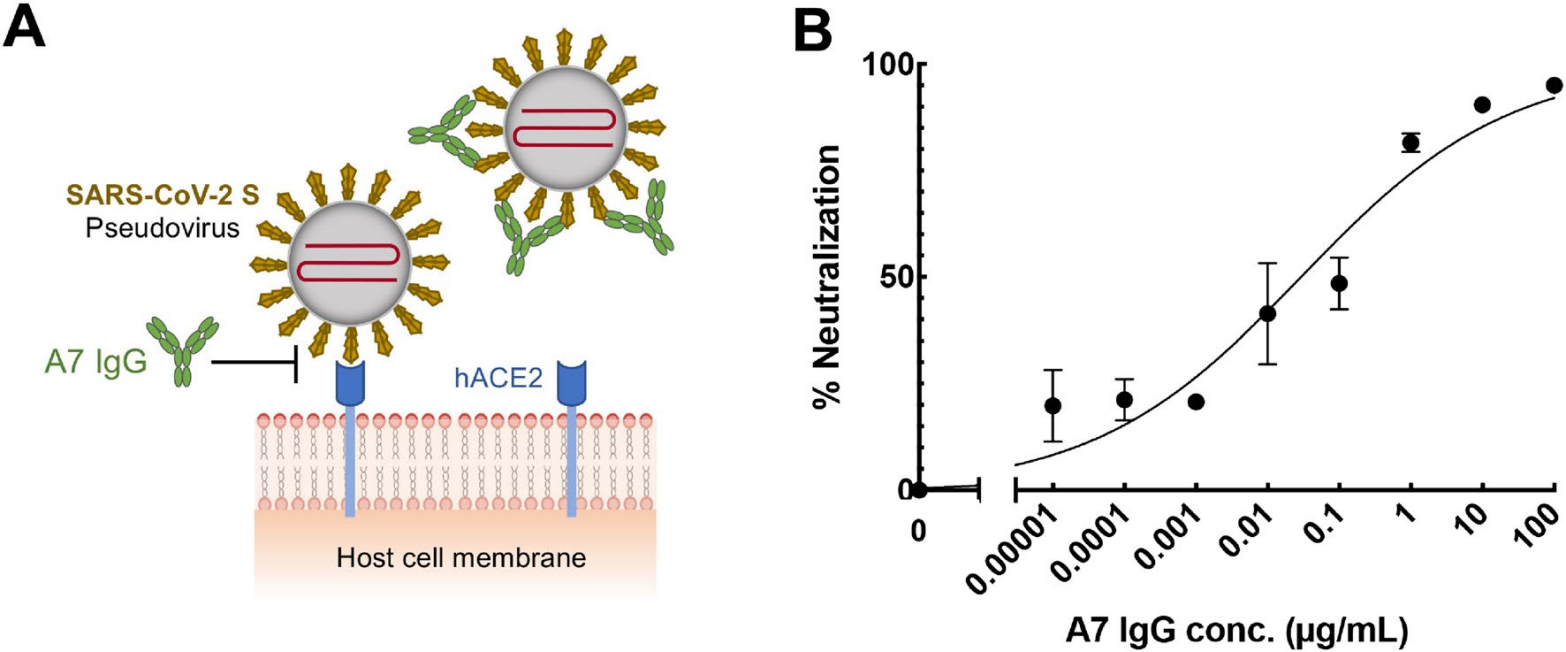
Neutralization of SARS-CoV-2 pseudovirus by A7 IgG. (**A**) Scheme and (**B**) results of neutralization of pseudovirus infection by A7 IgG. n = 3; Data have been expressed as mean ± standard deviation.

SARS-CoV-2 S pseudotyped human immunodeficiency virus simulates SARS-CoV-2 but has no toxicity. The pseudotyped virus can bind with ACE2 on the cell surface and initiate infection. The pseudovirus contains a luciferase gene, which it injects into the cells, to express luciferase, thus enabling detection of viral infection using the luminescent substrate luciferin. In this manner, the binding of the A7 antibody to the S protein and its neutralization activity can be detected by measuring the activity of intracellular luciferase, as shown in Fig. 6A. Different concentrations of A7 IgG were mixed with the virus, to infect cells. After culture, the cells were lysed, and their luciferase activity was determined by the addition of substrate. Figure 6B shows the neutralization effect of the A7 IgG. At low concentrations, A7 IgG did not neutralize the viral infection, but with an increase in concentration, the neutralization activity of the antibody increased gradually. An antibody concentration of 100 µg/mL was found to completely neutralize the viral infection. The inhibition constant IC_50_ for neutralization by A7 IgG was calculated as 0.03 µg/mL (0.2 nM) from the standard curve.

### Preparation of Q-body for virus detection

**Figure 7:**
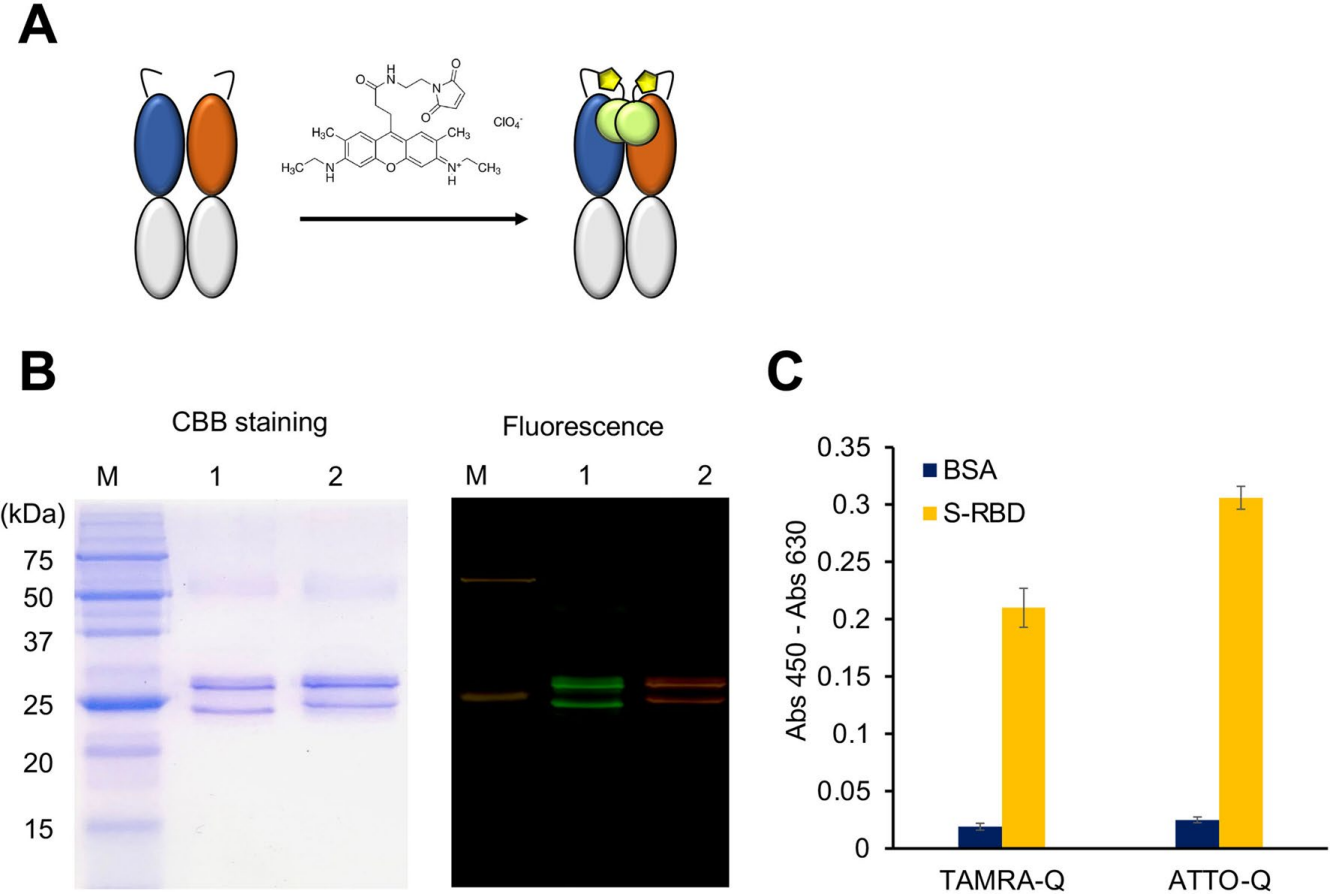
Conversion of A7 Fab to SARS-CoV-2-recognizing Q-body. (**A**) Scheme for preparation of Q-body; (**B**) CBB staining and fluorescent images of Q-body separated by SDS-PAGE. (**C**) Antigen-binding activities of TAMRA- and ATTO520-labeled Q-bodies. M: Precision Plus Protein™ Unstained Protein Standards spiked with 1% Dual-color Protein Standards (Bio-Rad); BSA: bovine serum albumin. n = 3; Data have been expressed as mean ± standard deviation. Original gel images are presented in Supplementary Fig. S4.

Since the clone A7 showed strong and specific binding to SARS-CoV-2 S protein, we attempted to make a fluorescent biosensor Q-body for rapid virus detection. To this end, the purified A7 Fab with two N-terminal Cys-containing tags was labeled with ATTO520 or TAMRA to prepare Q-bodies (Fig. 7A). The purification and fluorescence labeling of the Q-body were confirmed using SDS-PAGE. As shown in Fig. 7B, in the CBB-stained gel, two bands, one above and one below 25 kDa, were clearly observed. According to the theoretical molecular weight, the upper band was considered Fd, while the lower band was considered the light chain of A7 Fab. The gel was photographed using a fluorescein device before staining. It can be seen that the Fd and light chain of the antibody emit fluorescence, indicating that the fluorescence labeling was successful. The sample on the left is a Q-body labeled with ATTO520, while that on the right is a Q-body labeled with TAMRA.

The antigen-binding activity of Q-bodies was tested by ELISA. The absorbance at 450 nm for the TAMRA Q-body (TAMRA-Q) binding to S-RBD and BSA were 0.21 and 0.019, respectively, while the corresponding values for the ATTO520 Q-body (ATTO-Q) were 0.306 and 0.025, respectively. The results showed that both Q-bodies bound to S-RBD and retained the antigen-binding activity (Fig. 7C).

### Detection of SARS-CoV-2 spike protein trimer in saliva samples

**Figure 8:**
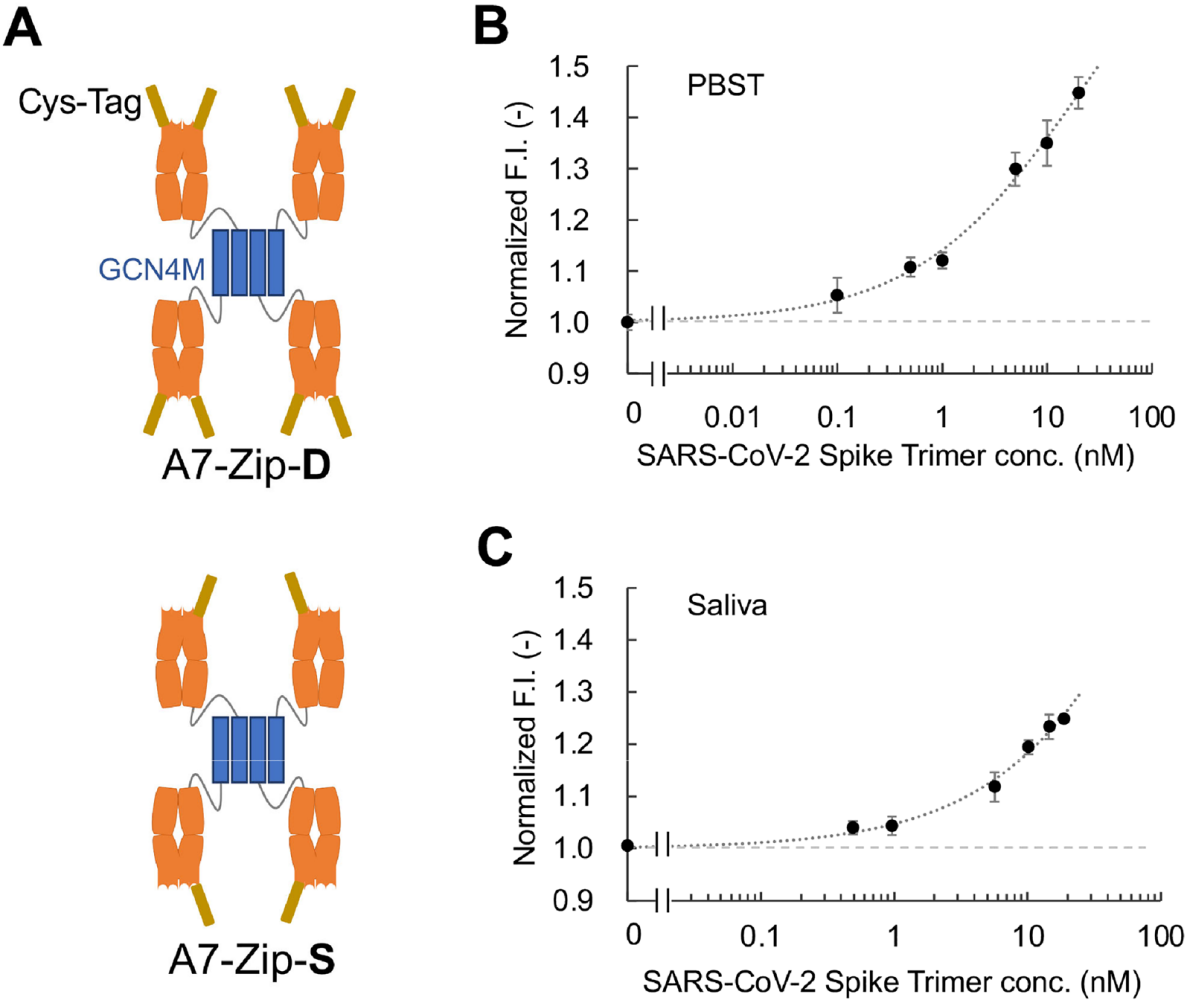
Oligomerized A7 Fab (A7-Zip) Q-body detects the SARS-CoV-2 spike protein at sub-nanomolar concentration. (**A**) Illustration of the two A7-Zip variants for Q-body conversion. A7-Zip-D: double dye-labeled on each Fab; A7-Zip-S: single dye-labeled on each Fab. The blue bar refers to the modified GCN4 oligomerization domain. (**B**) Dose–response curve for SARS-CoV-2 spike protein trimer spiked in PBST buffer with the A7-Zip-S-TAMRA Q-body. n = 4; (**C**) Dose–response curve for SARS-CoV-2 spike protein trimer spiked in human saliva sample with the A7-Zip-S-TAMRA Q-body. n = 3; Data have been expressed as mean ± standard deviation.

To increase the avidity to the polyvalent antigens such as S protein trimers and lower the detection limit, the A7 Fab was further engineered to produce two oligomerized variants, as shown in Fig. 8A. When the association and dissociation kinetics of A7-Zip-D against immobilized SARS-CoV-2 S1 was measured using BLI, the dissociation rate constant *k*_off_ (6.4 × 10^−5^/s) became approximately tenfold lower than that of A7 Fab. The *k*_on_ was 1.95 × 10^5^/Ms, which was similar to that of the A7 Fab, and the estimated *K*_D_ was 0.33 nM. Since the difference in avidity mainly contributed to the change in binding kinetics, the estimated *K*_D_ could not be compared with that of A7 Fab directly. The above two variants were labeled with TAMRA fluorescent dye for conversion to oligomerized Q-bodies, following which their responses against SARS-CoV-2-S1 and SARS-CoV-2-S-trimer were investigated. The TAMRA-labeled oligomerized Q-bodies showed 2–threefold higher response for S-trimer than that for S1 protein (Supplementary Fig. S2), with the limit of detection (LOD) for the S-trimer by A7-Zip-S-TAMRA Q-body (single dye-labeled on each Fab) in the sub-nanomolar range (0.11 nM) (Fig. 8B). The A7-Zip-S-TAMRA Q-body was also tested in human saliva samples spiked with S-trimer. After spiking with S-trimer, the reaction was incubated for 1 min and the fluorescence was measured using a tube fluorometer. An LOD of ~ 270 pM was achieved for spiked human saliva samples (Fig. 8C).

The responses of the A7-Zip-S-TAMRA Q-body against the spike proteins of four VOCs were also investigated (Supplementary Fig. S2). Due to mutations within the RBD of the spike protein mutants, the affinity of the A7 Fab against the mutants might be reduced (Fig. 2D,E). However, the LODs for the Alpha, Beta Gamma, and Omicron VOCs were determined as 0.81 nM, 0.55 nM, 2.2 nM and 3.5 nM, respectively, while two of which were still within the sub-nanomolar range.

### Detection of SARS-CoV-2 pseudovirus using Q-bodies

**Figure 9:**
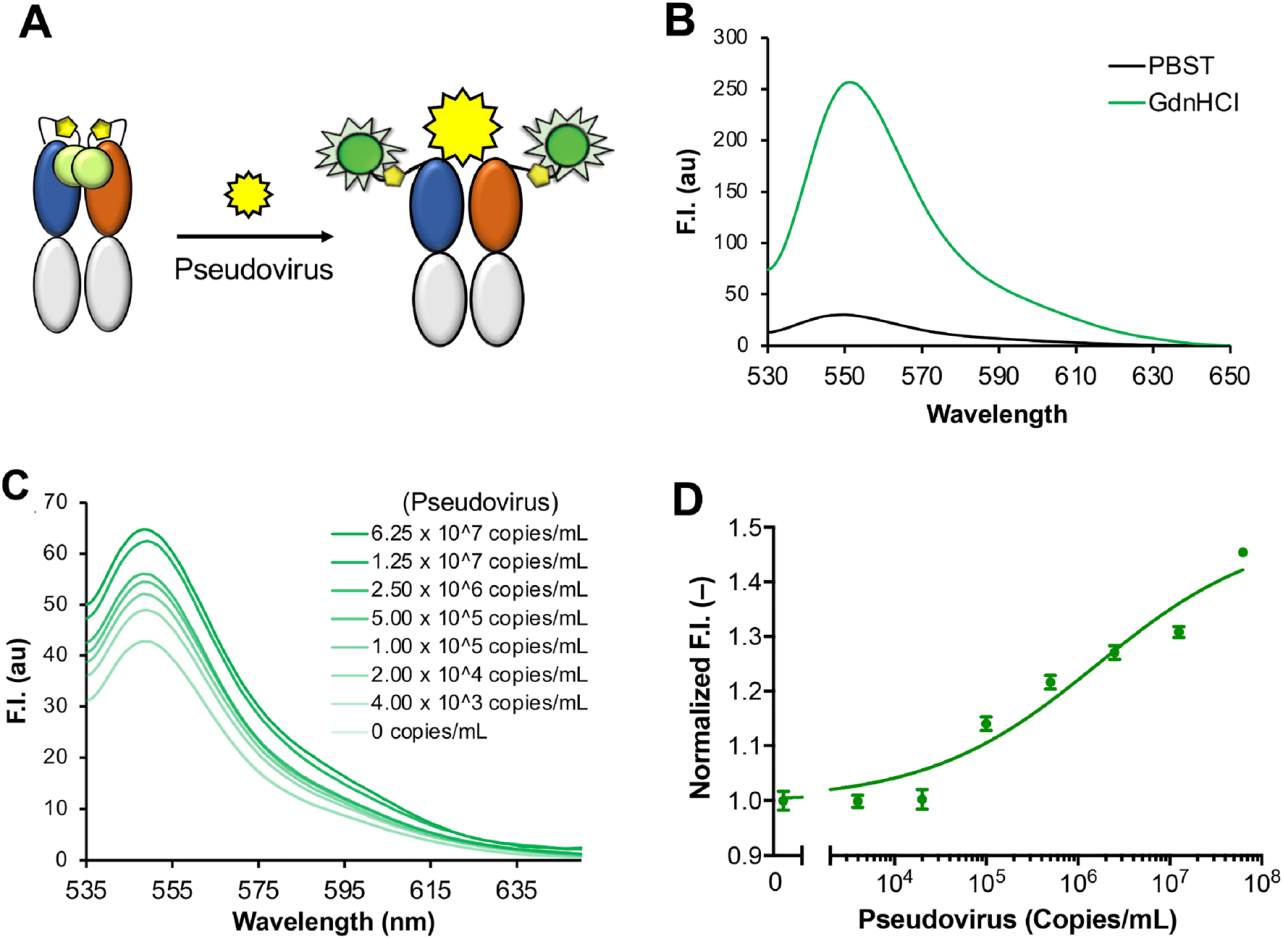
Detection of SARS-CoV-2 pseudovirus using a SARS-CoV-2-recognizing Q-body. (**A**) Scheme for detection of pseudovirus using Q-body; (**B**) Spectrum of Q-body in PBST and GdnHCl/DTT; (**C**) Fluorescence spectrum of Q-body for detection of SARS-CoV-2 pseudovirus; (**D**) Dose–response curve for detection of pseudovirus. n = 3; Data have been expressed as mean ± standard deviation.

We also attempted to detect S-pseudotyped virus particles using a double-labeled A7 Fab Q-body. As shown in Fig. 9A, when the double ATTO520-labeled Q-body binds to the pseudovirus, the quenched dyes will be de-quenched by the antigen-dependent removal of quenched H-dimers^21^. The fluorescence intensity of the Q-body solution will increase with the addition of S antigens on the surface; therefore, in reverse, the antigen can be detected by detecting the change in Q-body fluorescence intensity. In general, the degree of quenching of the Q-body can be evaluated by denaturing the Q-body. When Q-body was added to the guanidium hydrochloride (GdnHCl)/dithiothreitol (DTT) solution, the structure of the Q-body is completely denatured, and the fluorescence intensity of the labeled fluorescent dye will be completely restored. As shown in Fig. 9B, the maximum fluorescence intensity of the Q-body in PBS containing 0.1% Tween-20 (PBST) was 29.9, which increased 8.6-fold to 256.5 upon the addition of denaturant. After adding the pseudovirus to the Q-body solution and incubation for 2 min, the fluorescence intensity of the Q-body increased gradually, as shown in Fig. 9C. A standard curve was drawn with the maximum fluorescence intensity of each concentration, as shown in Fig. 9D. With the increase in virus concentration, the ratio of fluorescence increased gradually, and an LOD of 10^5^ copies/mL of pseudovirus was attained.

## Discussion

The development of SARS-CoV-2 antibodies mainly involves immunizing animals and extracting the blood of convalescent patients. These operations are tedious and time-consuming. In this study, antibodies were screened using the Tomlinson I human phage antibody library, resulting in the identification of four antibodies against the S protein of SARS-CoV-2. Clones A2 and A7 bound to S-RBD of S protein, while clones A3 and D4 bound to other domains of the S protein. We focused on clone A7 and prepared IgG and Fab variants for neutralizing viruses and making Q-bodies for detection of viruses, respectively. The *K*_D_ value of A7 Fab to S1 was 2.9 nM, which was strong enough to block the binding of ACE2 and SARS-CoV-2 S protein. When we changed the format to IgG, the apparent *K*_D_ value improved to 0.9 nM. It is worth noting that previous attempts using similarly obtained IgGs using the clone obtained from Tomlinson I library showed *K*_D_ values of more than 2 nM^37,38^. These results showed that our IgG can effectively block the infection of the pseudovirus, and thus, could be further investigated as a drug lead in future studies.

At present, the detection of SARS-CoV-2 is mainly based on reverse-transcription-polymerase chain reactions (RT-PCR), supplemented by antigen detection by LFA. The signal-on homogeneous antigen detection assay is an emerging tool with great potential in the virus diagnosis field because of its low cost, simple operation, and high throughput. Recently, a protein switch-based homogeneous assay for the detection of SARS-CoV-2 RBD was reported^39^. The reported LOD for the S-trimer was 0.05 nM, which was comparable to the oligomerized Q-body (0.11 nM), but this switch protein-based assay needs additional equilibration time after mixing all the components before adding the luciferase substrate. In our study, with the use of Q-bodies, 0.1 nM of S protein trimer or 10^5^ copies/mL of pseudovirus were successfully detected within 2 min, suggesting that the probe could realize rapid, accurate, and convenient real-time detection of SARS-CoV-2, to solve the shortcomings of current detection methods, such as longer detection periods, low throughput, and higher costs. The single-labeled oligomerized Q-body exhibited a better response against S-trimers, which could be used for developing the assay for the intact SARS-CoV-2 virus or concentrated spike protein antigen with the trimeric structure in the specimens. However, the yield of the oligomerized A7 Fab was about 10% of the Fab A7, and the relatively lower quenching efficiency increased the difficulty of improving the maximums response in the future. The double-labeled A7 Fab showed better quenching efficiency and the response could be further improved after linker or reaction buffer optimization. The nucleocapsid (N) protein is another important target for SARS-CoV-2 antigen test development^40^. The N protein is more abundant in nasopharyngeal swab samples within 7-days post symptom, which makes it easier to be detected in antigen tests without an additional concentration process^41,42^. Therefore, the Q-body against N protein will be developed in the following study as the complementary biosensor for the early phase infection diagnosis.

## Methods

### Materials

*Escherichia coli* XL10-Gold used for gene cloning and phagemid amplification was purchased from Agilent (La Jolla, CA, USA). The *E. coli* TG-1 strain used for phage production and Fab display was obtained from GE Healthcare (Tokyo, Japan). *E. coli *SHuffle T7 express and restriction enzymes used in this study were purchased from New England Biolabs (Ipswich, MA, USA), while the modification enzymes were purchased from Toyobo Biochemical (Osaka, Japan). The phage display library Tomlinson I and helper phage KM13 were obtained from Source BioScience, Nottingham, UK. SARS-CoV-2 S-pseudotyped human immunodeficiency virus PSV001 and the S protein fragments were obtained from Sino Bio Inc., Beijing, China, unless otherwise indicated. Other chemicals, reagents, and antibodies, unless otherwise indicated, were obtained from Sigma-Aldrich (St. Louis, MO, USA), Fujifilm Wako Pure Chemicals (Osaka, Japan), or Beijing Solarbio Science & Technology Co. Ltd. (Beijing, China). The primer sequences used in this study are listed in Supplementary Table S2. The primers used in this study were synthesized by Shanghai Sangon Biotech Co. Ltd. or Eurofins Japan (Tokyo, Japan).

### Screening of monoclonal antibody from a phage display library

Hundred microliters of PBS containing 10 µg/mL of SARS-CoV-2 S1 protein was added to 10 wells of a 96-well microplate and incubated overnight at 4 °C. The antigen solution was then discarded and 200 µL of PBS containing 2% skimmed milk (MPBS) was added to the wells. After incubation at 25 °C for 2 h, each well was washed three times with PBS containing 0.1% Tween 20 (PBST), before 100 µL of Tomlinson I library phage solution (R0; 10^9^ colony forming units; cfu per well) was added to each well and incubated at 25 °C for 2 h. After washing with PBST, 100 µL of 1 mg/mL trypsin was added to the wells, to elute the phage bound to the S1 protein.

*Escherichia coli* TG-1 was cultured to an optical density at 600 nm (OD_600_) of 0.4, following which 500 µL of the eluted phage solution was added to TG-1 to infect them, at 37 °C for 30 min. This was further cultivated at 37 °C overnight. Forty microliters of the overnight-cultivated *E. coli* TG-1 was then transferred to 4 mL 2YT medium (16 g/L tryptone, 10 g/L yeast extract, 5 g/L NaCl, pH 7.2) containing 100 µg/mL of ampicillin and 1% glucose (2YTAG), and cultured to an OD_600_ of 0.4. At this point, 10^10^ cfu of KM13 helper phage were added to the culture and it was allowed to incubate at 37 °C for 30 min. The cells were then pelleted by means of centrifugation at 5000×*g* for 20 min, following which the supernatant was discarded. The obtained pellet was re-suspended in 4 mL of 2YT medium containing 100 µg/mL of ampicillin, 50 µg/mL kanamycin, and 0.1% glucose (2YTAK), and cultivated for 16 h at 30 °C, with shaking at 250 rpm. The overnight culture was centrifuged at 5000×*g* for 30 min, following which the supernatant was collected, and 1/5th volume of 20% polyethylene glycol 6000/2.5 M NaCl solution was added to it, mixed well, and placed on ice for 30 min. Centrifugation was carried out at 5000×*g* for 30 min, and the pellet was resuspended in 200 µL of sterile PBS solution, which was stored as R1 phage solution. The above steps were repeated to obtain an R2 phage solution as well.

To obtain monoclonal phages, *E. coli* TG-1 was cultured to an OD_600_ of 0.4, and 100 µL of R2 phage antibody library solution was used to infect 200 µL of *E. coli* TG-1, which were then plated on Luria–Bertani (LB) medium (BD Biosciences, Franklin Lakes, NJ, USA) plates containing 100 µg/mL ampicillin and 1% glucose, before culturing overnight at 37 °C. Ninety-six colonies in 100 µL 2YTAG medium each were inoculated into wells of a 96-well microplate and allowed to grow at 37 °C, until the OD_600_ reached 0.4, at which point, KM13 helper phage (5.2 × 10^10^ plaque forming units; pfu) was added into each well. After infection, the cells were pelleted by means of centrifugation at 5000×*g* for 20 min, the obtained supernatant was discarded, and the cell pellet was re-suspended in 200 µL of 2YTAK. The suspension cells were cultured for 16 h at 250 rpm/30 °C. Centrifugation was performed again to recover the supernatant as a phage solution, following which ELISA was performed to verify the antigen-binding affinity and specificity of each clone.

### ELISA

ELISA was carried out to verify the binding specificity and performance of the phage display antibody library obtained during the bio-panning or of an individual clone against the S1 protein.

ELISA was performed as follows: 100 µL PBS solution containing S1 protein, S-RBD protein (GenScript Inc., Nanjing, China; 2 µg/mL), or BSA (2 µg/mL) was added to a 96-well microplate, and the antigen solution was left overnight at 4 °C. MPBS (200 µL) was then added to the cells and the mixture was incubated at 25 °C for 2 h. The plate was washed with PBST three times, following which 10^9^ cfu/well of R0, R1, and R2 phage solutions or individual phage clones were added to the wells.

After incubation at 25 °C for 1 h, the plate was washed with PBST solution, and the 1/5000-diluted HRP/anti-M13 antibody (GE Healthcare, Tokyo, Japan) in PBST was added to the wells. After incubation for 1 h, the plate was washed with PBST again, and 100 µL of TMBZ (0.2 mg/mL in 100 mM of sodium acetate at pH 6.0 with 1:10,000 diluted 30% H_2_O_2_) was added to the wells, and the assay was developed. The absorbance of the wells was measured at the wavelength of 450 nm using an iMark™ microplate reader (Bio-Rad, Heracules, CA, USA) after terminating the reaction with 50 µL of 10% H_2_SO_4_.

ELISA was also carried out to confirm the antigen-binding activity of the purified Fab fragments using the same protocol as above, except that in this case, after blocking and washing, purified Fab in PBS (at a concentration of 5 µg/mL) was added unless otherwise stated. After incubation for 1 h at 25 °C, HRP-conjugated anti-His mouse monoclonal antibody (BBI Life Sciences Co., Shanghai, China) was added to the wells.

Antigen information and modified conditions of the ELISA for verifying the affinity of Fab A7 against full-length spike proteins are listed below. Antigens were coated on the plate at the concentration of 5 µg/ml spike proteins (Spike protein of wild-type SARS-CoV-2, 10549-CV-100, R&D Systems; Spike protein of SARS-CoV-2 previous variant of concern alpha, 40589-V08B6, Sino Biological; Spike protein of SARS-CoV-2 previous variant of concern beta, 40589-V08B7, Sino Biological; Spike protein of SARS-CoV-2 previous variant of concern gamma, 40589-V08B8, Sino Biological; Spike protein of SARS-CoV-2 variant of concern delta, 40589-V08B16, Sino Biological; Spike protein of SARS-CoV-2 variant of concern omicron, 40589-V08H26, Sino Biological). A7 Fab concentration was 20 nM and the secondary antibody was 1/10,000-diluted in the assay (monoclonal Anti-FLAG M2-Peroxidase, A8592).

### Expression and purification of Fab fragments

PCRs were performed to amplify the variable region of the heavy chain (V_H_) gene with the primers AgeInCoV2no1Vhback/XhoInCoV2no1VHfor and that of the light chain (V_L_) with the primers SpeICoV2no1Vlback/HindIIInCoV2no2Vlfor, while using the screened antibody plasmid as a template. Each reaction was performed in a volume of 50 µL, with the following reaction conditions: denaturation at 94 °C for 2 min, 30 cycles of denaturation at 94 °C for 30 s, 55 °C for 30 s, and 68 °C for 1 min. Subsequently, the products were detected using 1% agarose gel, and the target gene fragments were recovered. After purification, the V_L_ fragments were treated with the restriction enzymes *Spe*I*/Hin*dIII. After gel purification, the fragment was ligated to the pUQ2 vector treated with the same enzymes using Ligation High Ver. Two, and then transformed into *E. coli* XL10-Gold. The insert in the plasmid was confirmed using colony PCR, following which the clones containing the gene fragments were cultivated to extract plasmids for sequence analysis. The gene fragments for V_H_ were also amplified and cloned into pUQ2 containing the cloned V_L_ after treatment with *Age*I/*Xho*I.

*Escherichia coli* SHuffle T7 express was transformed with the constructed plasmid and cultured on LB medium containing 100 µg/mL ampicillin (LBA) agar (1.5%) plates at 37 °C for 14 h. The single colonies obtained were then selected and inoculated into 4 mL liquid LBA medium. After overnight culture at 37 °C with shaking at 200 rpm, the culture was transferred to 100 mL LBA. When the OD_600_ reached 0.4, isopropyl-β-d-thiogalactoside was added to the culture at a final concentration of 0.4 mM, to induce protein expression, and the culture was allowed to grow for an additional 18 h at 16 °C with shaking.

The bacterial culture was centrifuged at 6000×*g* for 20 min, following which the obtained pellet was re-suspended in TALON extraction buffer (8 mM Na_2_HPO_4_, 47.9 mM NaH_2_PO_4_, and 300 mM sodium chloride, pH 7.0), and the cells were lysed by means of sonication. The cell lysate was then subjected to centrifugation, to separate and collect the supernatant, from which the Fab was extracted and purified using TALON beads, according to the manufacturer’s protocol. SDS-PAGE was performed to analyze the purity of Fab^43^. The concentration of Fab was determined using Bradford assay (Thermo Fisher Scientific, Waltham, MA, USA).

### ELISA-based neutralization assay

ELISA was performed to test the neutralization function of the A7 Fab. Recombinant ACE2 (ab151852, Abcam, Cambridge, UK) was immobilized on a microplate at a concentration of 2 µg/mL. After overnight incubation at 4 °C, the solution was removed and the microplate was blocked with 20% (v/v) ImmunoBlock (KAC, Hyogo, Japan) in PBST. After 2 h, the plates were washed again and 10 nM biotin-SARS-CoV-2 S1 protein (40591-V08H-B, Sino Biological Inc., Beijing, China) with purified A7 Fab antibody fragment (100 µL, at concentrations of 0.1–5000 nM, pre-incubated for 1 h at room temperature) was added into each well, while only 10 nM biotin-S1 protein was added in the control group. After incubation for 2 h, the plate was washed three times with PBST, followed by the addition of 100 µL Neutravidin-HRP (1:1000 dilution in PBST containing 5% ImmunoBlock). After 1 h of incubation and washing, 100 µL of substrate solution (0.2 mg/mL TMBZ and 30 mM H_2_O_2_ in 100 mM sodium acetate, pH 6.0) was added to develop the assay, and the absorbance at 450 nm and 655 nm was measured by a microplate reader SH-1000 (Corona Electric, Ibaraki, Japan) after terminating the reaction with 50 µL of 10% H_2_SO_4_. The inhibition constant *K*_i_ was estimated according to the Eq. (1).

1 $$\text{IC}_{50} = K_\text{i} \times (1+\frac{[\text{S}1]}{{K}_{\text{D }(\text{ACE2 against S1})}})$$

### Preparation of Q-body for virus detection

Tris[2-carboxyethyl] phosphine hydrochloride was added to 100 µL of 10 µM A7 Fab solution until a final concentration of 0.5 mM, and incubated with rotation for 20 min in the dark, followed by the addition of 4-azidobenzoic acid solution at a final concentration of 2 mM. After incubation of the reaction solution on ice for 10 min, ATTO520-maleimide (ATTO-TEC GmbH, Siegen, Germany) or 5-TAMRA-C6-maleimide (AAT Bioquest, Sunnyvale, CA, USA) was added to the reaction mixture at a final concentration of 1 mM and rotated for 2 h in the dark. Twenty microliters of pre-treated anti-FLAG magnetic beads (M8823, Sigma-Aldrich) were added to the tube and incubated for 2 h in the dark. The magnetic beads were washed 11 times with PBS containing 0.1% Brij35, and three times with PBST. The labeled A7 Fab (A7 Q-body) was eluted with 100 µL of 150 µg/mL 3 × FLAG peptide in PBST. The purity and fluorescence of the A7 Q-body were analyzed using SDS-PAGE and ELISA. To measure the quenching efficiency, 1 µL of A7 Q-body was added to 800 µL of PBST or 7 M GdnHCl/0.5 M DTT, and the fluorescence spectra and values were measured using a Hitachi 4600 spectrophotometer or CLARIOstar multiplate reader (BMG Labtech, Ortenberg, Germany), respectively. SARS-CoV-2 pseudovirus was added to A7 Q-body solution at the final concentrations of 0, 4.0 × 10^3^, 2.0 × 10^4^, 1.0 × 10^5^, 5.0 × 10^5^, 2.5 × 10^6^, 1.25 × 10^7^, and 6.25 × 10^7^ copies/mL from the stock solution (10^10^ copies/mL). The control group was treated with the same volume of Dulbecco’s modified Eagle medium instead of the pseudovirus and detected under the same conditions.

To improve the response speed and sensitivity of the spike protein trimer, oligomerized A7 Q-bodies were also tested. The oligomerized Fab A7 (A7-Zip-D) was constructed by adding a hinge region and a modified GCN4 zipper (GCN4M) sequence at the C-terminal of Fd^44^. Briefly, the pUQ2-A7 plasmid was linearized by means of inverse PCR using the primers BZ-UQ2-VHendBreak-F and BZ-UQ2-VHendBreak-R. The DNA fragment of GCN4M was prepared by means of hybridization and extension of two oligo DNAs, BZ-oligo-GCN4M-F and BZ-oligo-GCN4M-F, and then inserted into the linearized pUQ2-A7 via In-Fusion DNA assembly (Takara Bio, Shiga, Japan) to obtain the plasmid pUQ2-A7-Zip-D. A single Cys-tag variant of oligomerized Fab A7 (A7-Zip-S) was constructed by removing the Cys-tag from the L chain of A7-Zip-D. The plasmid pUQ-A7-Zip-S was constructed by means of inverse PCR using the primers BZ-pUQ2-CysR-InvF2 and BZ-UQ2A7-CysR-InsR, with pUQ2-A7-Zip-D as the template, followed by self-ligation using In-Fusion® DNA assembly. The A7-Zip-D Q-body and A7-Zip-S Q-body were prepared by labeling with ATTO520 or TAMRA, as described above. Their responses against SARS-CoV-2 S1 protein (40591-V08H, Sino Biological Inc.) and spike protein trimer (extra-cellular domain, ECD, 10549-CV-100, R&D Systems, Minneapolis, MN, USA) were investigated in PBST buffer or human saliva samples. The Q-body concentration (calculated based on a monomeric Fab unit) was 0.5 nM for spiked PBST and 2 nM for spiked saliva samples. To mimic the on-site application of the Q-body immunosensor, 200 µL of human saliva sample (n = 3) was spiked with different concentrations of spike trimer protein, and the fluorescence intensity was measured after mixing for 1 min and incubation at room temperature using a tube-based fluorometer (DeNovix DS11 equipped with FX module). A green LED with a wavelength of 525 nm and an emission filter of 565–650 nm were used for signal detection. A blank sample (Q-body in saliva) spiked with PBST was used to normalize the fluorescence intensity. Dose–response curves were fitted to a four-parameter logistic Eq. (2) using Prism 8 (GraphPad Software, San Diego, CA, USA) or SciDAVis software (version 2.4.0, http://scidavis.sourceforge.net/) with “a” set to 1 as a constant parameter. The LOD was calculated as the concentration corresponding to the mean blank value plus three times the standard deviation.

2 $$y = d+\frac{a-d}{1+{\left(\frac{x}{c}\right)}^{b}}$$

### Construction of vectors for expression of full-length A7 IgG

The gene for V_H_ of A7 was amplified using KOD-Plus-Neo DNA polymerase (Toyobo Biochemical) with pIT2-A7 as the template and the primers InfuEcoRISARS-CoV-2-A7VHback and InfuNheIXhoInCoV-2-A7VHfor. PCR was carried out for 30 cycles, with each cycle consisting of denaturation at 94 °C for 2 min, annealing at 55 °C for 30 s, and extension at 68 °C for 1 min, followed by denaturation at 94 °C for 2 min. The PCR product was purified and cloned into *Eco*RI/*Nhe*I-digested expression vector pDongIgG1HChn, which was made from pFUSE-hIgG1-Fc2 (InvivoGen, Pak Shek Kok, Hong Kong) by the addition of hIgG1 C_H_1 gene, *Eco*RI/*Nhe*I enzyme sites for cloning V_H_, and destruction of the original *Nhe*I site of the plasmid, using the ClonExpress II One Step Cloning Kit (Vazyme, Nanjing, China), according to the manufacturer’s instructions. After overnight culture, colony PCR was performed to screen for colonies containing antibody genes, followed by confirmation of the sequence. The resultant vector for expression of the A7 Fd chain was named pDongHuG1c-A7. The gene for A7 V_L_ was amplified using pIT2-A7 as a template and the primers InfuEcoRISARS-CoV-2-A7VLback and BsiWISARS-CoV-2-A7VLfor. The Cκ gene was amplified using KOD-Plus-Neo with pFUSE2-CLIg-hk as the template and the primers OverlapCKBack and InfusionNheICKFor. V_L_ and Cκ were fused by means of overlap PCR and cloned into the *Eco*RI/*Nhe*I-treated pFUSE-hIgG1-Fc2 vector as described above, resulting in pDongHukc-A7.

### Preparation of A7 IgG

The frozen HEK293F cells were resuscitated at 37 °C, added into a 125 mL flask containing 20 mL serum-free medium 293 T II (Sino Biological Inc.), and incubated in an incubator with 8% CO_2_ at 37 °C, with shaking at 120 rpm. The cells were adjusted to a density of 1.5 × 10^6^ cells/mL in serum-free medium and cultivated for 2 h with shaking. Ten micrograms of the vectors for the expression of the antibody heavy and lights expression were mixed with 40 µL of Lipofectamine™ 8000 transfection reagent (Beyotime Biotechnology Inc., Nanjing, China) and added to 1 mL serum-free medium. The plasmids were gently mixed and incubated at 37 °C for 15 min. The transfection reagent-DNA mixture was then slowly added into the flask with gentle shaking, to ensure that it is evenly dispersed into the cell culture medium. The cells were cultured for 48 h and centrifuged at 1000 rpm for 5 min, to obtain the supernatant that consisted of full-length A7 IgG.

The antibody was purified using Protein A magnetic beads (GenScript Inc, Nanjing, China). Briefly, 100 μL of Protein A beads were taken, washed three times with binding/wash buffer, and collected using a magnetic rack. The magnetic beads were resuspended in A7 IgG solution and mixed for 60 min at 25 °C on a rotator. The magnetic beads were collected using a magnetic rack and washed three times. Hundred microliters of elution buffer was added to the collected beads and incubated at 25 °C for 5 min, during which the solution was mixed several times. The magnetic beads were collected using a magnetic rack, following which, the supernatant containing eluted IgG was transferred to a new centrifuge tube, neutralized by the addition of 10 μL neutralization buffer, and stored for subsequent analysis. ELISA was carried out to confirm the antigen-binding activity, in which IgG against RBD (40150-D001) and S1(40150-R007) from Sino Biological Inc. were used as positive controls.

### Determination of antigen-binding affinities

Kinetic parameters for the Fabs and their oligomerized variants, as well as the IgG derived from clone A7 were analyzed using a BLI system equipped with a streptavidin (SA) sensor on the Octet K2 and Octet Red 96e systems (Pall FortéBio Corp., Menlo Park, CA, USA), respectively. The SA probe was treated with PBS buffer containing 2% BSA for 10 min and loaded with biotinylated S-proteins (SARS-CoV-2 S1, 40591-V08H-B; SARS-CoV S1, 40150-V08B1-B; SARS-CoV-2 RBD, 40592-V08H-B; Sino Biological Inc.) at concentrations of 1 µM or 100 nM. A7 Fab was applied at concentrations ranging from 10 to 200 nM for different assays, and A7-IgG was applied at concentrations of 25 nM, 50 nM, 100 nM, 200 nM, and 400 nM, following which the kinetic parameters were estimated using Data Analysis 8.1 HD (Pall FortéBio Corp.). Similarly, the affinity between ACE2 and S1 protein was measured by loading the ACE2 protein on the AR2G biosensors according to the manufacturer's protocol, and the 50 nM and 100 nM S1 protein are used for the affinity measurement.

### Neutralization of infection by A7 IgG

The gene for human ACE2 cDNA was amplified from pcDNA6B-ACE2 (MiaolingBio, Wuhan, China) using the primers hACE2CDSFor and hACE2CDSRev and inserted into pcDNA3.1-EGFP-C (YouBio, Wuhan, China), between the *Eco*RI and *Xho*I restriction sites, to construct pcDNA3.1 ( +)-ACE2-EGFP, for transient expression of ACE2-EGFP. 293 T cells cultured in DMEM containing 10% fetal bovine serum and 100 U/mL penicillin and streptomycin were transfected with pcDNA3.1 ( +)-ACE2-EGFP using Lipofectamine™ 8000, to prepare ACE2-overexpressing cells. Ten microliters of SARS-CoV-2 S-spiked pseudovirus PSV001 (Sino Biological Inc.; 10^10^ virus copies/mL) were mixed with different concentrations of A7 IgG in 90 µL PBS, at final concentrations from 10 pg/mL to 10 µg/mL, followed by incubation at 37 °C for 1 h. The mixtures were added to ACE2-overexpressing cells, cultured for 24 h, replaced with fresh medium, and cultured for another 48 h. Following that, the cells were lysed and their intracellular luciferase activity was measured using a Spark® multimode microplate reader (TECAN, Shanghai, China) with a Firefly Luciferase Reporter Gene Assay Kit (Beyotime Biotechnology Inc.), according to the manufacturer’s instructions. The viral stock solutions (10^8^ virus copies) were used as a positive control group, and neutralization efficiency was calculated as the decrease in luminescence, as compared to that of the positive controls. The detection was performed in triplicate.

## Supplementary Information

Supplementary Information.

## Data Availability

All data generated during this study are included in this published article and its supplementary information files.

## Abbreviations

ACE2: Angiotensin-converting enzyme 2
CDRs: Complementarity-determining regions
ECD: Extracellular domain
ELISA: Enzyme-linked immunosorbent assay
Fab: Antigen-binding fragment
HRP: Horseradish peroxidase
IgG: Immunoglobulin G
LOD: Limit of detection
Q-body: Quenchbody
RBD: Receptor-binding domain
SA: Streptavidin
SARS-CoV-2: Severe acute respiratory syndrome coronavirus 2
scFv: Single-chain variable fragment
5-TAMRA: 5-Carboxytetramethylrhodamine
TCEP: Tris(2-carboxyethyl)phosphine
VOC: Variants of concern

## References

[1] Zhou P (2020). A pneumonia outbreak associated with a new coronavirus of probable bat origin. Nature.

[2] Walls AC (2020). Structure, function, and antigenicity of the SARS-CoV-2 spike glycoprotein. Cell.

[3] Hoffmann M (2020). SARS-CoV-2 cell entry depends on ACE2 and TMPRSS2 and is blocked by a clinically proven protease inhibitor. Cell.

[4] Yan R (2020). Structural basis for the recognition of SARS-CoV-2 by full-length human ACE2. Science.

[5] Lan J (2020). Structure of the SARS-CoV-2 spike receptor-binding domain bound to the ACE2 receptor. Nature.

[6] Li W (2003). Angiotensin-converting enzyme 2 is a functional receptor for the SARS coronavirus. Nature.

[7] Lu R (2020). Genomic characterisation and epidemiology of 2019 novel coronavirus: Implications for virus origins and receptor binding. Lancet.

[8] Drozdzal S (2020). FDA approved drugs with pharmacotherapeutic potential for SARS-CoV-2 (COVID-19) therapy. Drug Resist. Update.

[9] Artese A (2020). Current status of antivirals and druggable targets of SARS CoV-2 and other human pathogenic coronaviruses. Drug Resist. Update.

[10] Baum A (2020). REGN-COV2 antibodies prevent and treat SARS-CoV-2 infection in rhesus macaques and hamsters. Science.

[11] Jiang S, Hillyer C, Du L (2020). Neutralizing antibodies against SARS-CoV-2 and other human coronaviruses. Trends Immunol..

[12] Oliviero A, de Castro F, Coperchini F, Chiovato L, Rotondi M (2020). COVID-19 pulmonary and olfactory dysfunctions: Is the chemokine CXCL10 the common denominator?. Neuroscientist.

[13] Wu Y (2020). Identification of human single-domain antibodies against SARS-CoV-2. Cell Host Microbe.

[14] Huo J (2020). Neutralizing nanobodies bind SARS-CoV-2 spike RBD and block interaction with ACE2. Nat. Struct. Mol. Biol..

[15] Barnes CO (2020). SARS-CoV-2 neutralizing antibody structures inform therapeutic strategies. Nature.

[16] Hansen J (2020). Studies in humanized mice and convalescent humans yield a SARS-CoV-2 antibody cocktail. Science.

[17] Monteil V (2020). Inhibition of SARS-CoV-2 infections in engineered human tissues using clinical-grade soluble human ACE2. Cell.

[18] Cardone M, Yano M, Rosenberg AS, Puig M (2020). Lessons learned to date on COVID-19 hyperinflammatory syndrome: Considerations for interventions to mitigate SARS-CoV-2 viral infection and detrimental hyperinflammation. Front. Immunol..

[19] Iwanaga N (2020). Novel ACE2-IgG1 fusions with improved activity against SARS-CoV2. bioRxiv.

[20] Abe R (2011). "Quenchbodies": Quench-based antibody probes that show antigen-dependent fluorescence. J. Am. Chem. Soc..

[21] Abe R (2014). Ultra Q-bodies: Quench-based antibody probes that utilize dye-dye interactions with enhanced antigen-dependent fluorescence. Sci. Rep..

[22] Ohashi H (2016). Insight into the working mechanism of quenchbody: Transition of the dye around antibody variable region that fluoresces upon antigen binding. Bioconjug. Chem..

[23] Zhao S, Dong J, Jeong HJ, Okumura K, Ueda H (2018). Rapid detection of the neonicotinoid insecticide imidacloprid using a quenchbody assay. Anal. Bioanal. Chem..

[24] Inoue A, Ohmuro-Matsuyama Y, Kitaguchi T, Ueda H (2020). Creation of a nanobody-based fluorescent immunosensor mini Q-body for rapid signal-on detection of small hapten methotrexate. ACS Sens..

[25] Dong J, Fujita R, Zako T, Ueda H (2018). Construction of Quenchbodies to detect and image amyloid beta oligomers. Anal. Biochem..

[26] Jeong HJ (2017). Development of a quenchbody for the detection and imaging of the cancer-related tight-junction-associated membrane protein Claudin. Anal. Chem..

[27] Jeong HJ, Dong J, Ueda H (2018). Single-step detection of the influenza virus hemagglutinin using bacterially-produced Quenchbodies. Sensors.

[28] Dong J, Oka Y, Jeong HJ, Ohmuro-Matsuyama Y, Ueda H (2020). Detection and destruction of HER2-positive cancer cells by Ultra Quenchbody-siRNA complex. Biotechnol. Bioeng..

[29] Jeong HJ (2013). Detection of vimentin serine phosphorylation by multicolor Quenchbodies. Biosens. Bioelectron..

[30] Dong J, Jeong HJ, Ueda H (2016). Preparation of Quenchbodies by protein transamination reaction. J. Biosci. Bioeng..

[31] Jeong HJ (2017). Construction of dye-stapled Quenchbodies by photochemical crosslinking to antibody nucleotide-binding sites. Chem. Commun. (Camb.).

[32] Dong J (2020). PM Q-probe: A fluorescent binding protein that converts many antibodies to a fluorescent biosensor. Biosens. Bioelectron..

[33] Smith GP (1985). Filamentous fusion phage: Novel expression vectors that display cloned antigens on the virion surface. Science.

[34] McCafferty J, Griffiths AD, Winter G, Chiswell DJ (1990). Phage antibodies: Filamentous phage displaying antibody variable domains. Nature.

[35] de Wildt RMT, Mundy CR, Gorick BD, Tomlinson IM (2000). Antibody arrays for high-throughput screening of antibody–antigen interactions. Nat. Biotechnol..

[36] Swindells MB (2017). abYsis: Integrated antibody sequence and structure: Management, analysis and prediction. J. Mol. Biol..

[37] Parray HA (2020). Identification of an anti-SARS-CoV-2 receptor-binding domain-directed human monoclonal antibody from a naive semisynthetic library. J. Biol. Chem..

[38] Yuan M (2021). Identification and characterization of a monoclonal antibody blocking the SARS-CoV-2 spike protein-ACE2 interaction. Cell Mol. Immunol..

[39] Quijano-Rubio A (2021). De novo design of modular and tunable protein biosensors. Nature.

[40] Grant BD (2020). SARS-CoV-2 coronavirus nucleocapsid antigen-detecting half-strip lateral flow assay toward the development of point of care tests using commercially available reagents. Anal. Chem..

[41] Pollock NR (2021). Correlation of SARS-CoV-2 nucleocapsid antigen and RNA concentrations in nasopharyngeal samples from children and adults using an ultrasensitive and quantitative antigen assay. J. Clin. Microbiol..

[42] Shan D (2021). N-protein presents early in blood, dried blood and saliva during asymptomatic and symptomatic SARS-CoV-2 infection. Nat. Commun..

[43] Laemmli UK (1970). Cleavage of structural proteins during the assembly of the head of bacteriophage T4. Nature.

[44] Pack P, Müller K, Zahn R, Plückthun A (1995). Tetravalent miniantibodies with high avidity assembling in *Escherichia coli*. J. Mol. Biol..

