# Supplementary material for: Isolation of a human SARS-CoV-2 neutralizing antibody from a synthetic phage library and its conversion to fluorescent biosensors
Source: Sci Rep. 2022 Sep 15;12:15496. doi: 10.1038/s41598-022-19699-z (PMC9476436; doi:10.1038/s41598-022-19699-z)
Supplement: Supplementary file 1 — Supplementary Information. [file 41598_2022_19699_MOESM1_ESM.pdf]

Supplementary Information: Isolation of a Human SARS-CoV-2 Neutralizing Antibody from a Synthetic Phage Library and its Conversion to Fluorescent Biosensors

Haimei Li[1#], Bo Zhu[2#], Baowei Li[1], Limei Chen[1], Xuerao Ning[2], Hang Dong[3], Jingru Liang[1], Xueying Yang[1], Jinhua Dong[1,2,4*], Hiroshi Ueda[2,4*]

[1]Weifang Key Laboratory for Antibodies Medicine, School of Life Science and Technology, Weifang Medical University, Weifang, China
[2]Laboratory for Chemistry and Life Science, Institute of Innovative Research, Tokyo Institute of Technology, Yokohama, Japan
[3]School of Basic Medical Sciences, Peking University, Beijing, China
[4]World Research Hub Initiative (WRHI), Institute of Innovative Research, Tokyo Institute of Technology, Yokohama, Japan

[#]These authors contributed equally.
[*]Contacts
Jinhua Dong : dongjh@wfmc.edu.cn Weifang Key Laboratory for Antibodies Medicine, School of Life Science and Technology, Weifang Medical University, Weifang 261053, China
Hiroshi Ueda : ueda@res.titech.ac.jp Laboratory for Chemistry and Life Science, Institute of Innovative Research, Tokyo Institute of Technology, Nagatsuta-cho, Midori-ku, Yokohama 226-8503, Japan

Supplementary Tables S1-S2
Supplementary Figures S1-S3

Supplementary Table S1. Summary of kinetics analysis for the obtained antibodies

Antibody	Ligand	K_D (nM)	k_{on}(10^5/Ms)	k_{off} (10^{-4}/s)	Full R^2	Analysis Note
Fab-A7	CoV-2-S1	2.89	1.86	5.38	0.960	Global fitting
	CoV-2-RBD	7.42	0.809	6.00	0.996	Global fitting
	CoV-1-S1	31.1	0.542	16.9	0.887	Global fitting
Fab-A2	CoV-2-S1	52.8	0.269	14.2	0.904	Global fitting
	CoV-2-RBD	9.92	0.752	7.46	0.967	Global fitting
Fab-A3	CoV-2-S1	43.9	0.334	14.7	0.912	Local fitting
	CoV-2-RBD	n.d.	n.d.	n.d.		
A7 IgG	CoV-2-RBD	0.937	2.96	2.78	0.989	Global fitting

CoV-2-S1: SARS-CoV-2 Spike S1 (HEK293-derived, Biotinylated, Sino Biological, Cat. 40591-V08H-B)

CoV-2-RBD: SARS-CoV-2 Spike RBD (HEK293-derived, Biotinylated, Sino Biological, Cat. 40592-V08H-B)

CoV-1-S1: SARS-CoV Spike S1 (Baculovirus-insect cells-derived, Biotinylated, Sino Biological, Cat: 40150-V08B1-B)

n.d.: Not detected due to low regression reliability. $R^2 < 0.95$ are colored in blue.

Supplementary Table S2. Sequence of DNA oligos used in this study

**Name**	**Sequence (5´ to 3´)**
SpeICoV2no1VLback	TGAGACTAGTGACATCCAGATGACCCAGT
HindIIInCoV2no2VLfor	TTTCAAGCTTGGTCCCTTGGCCGAACGTA
AgeInCoV2no1VHback	GAGACCGGTGAGGTGCAGCTGTTGGA
XhoInCoV2no1VHfor	AGCGCTCGAGACGGTGACCAGGGTTCCCT
BZ-oligo-GCN4M-F	CTGAATGGGGCCGCACCGAAACCGAGCACCCCGC CGGGCAGCAGCCGCCTGAAACAGATTGAAGATAA ACTGGAAGAAATTCTGAGCAAAC
BZ-oligo-GCN4M-R	CCTTCTAGATTATTAGCGTTCGCCCAGCAGTTTTTT AATGCGCGCCAGTTCGTTTTCAATATGATACAGTT TGCTCAGAATTTCTTCCAG
BZ-UQ2-VHendBreak-F	TAATAATCTAGAAGGAGATATC
BZ-UQ2-VHendBreak-R	TGCGGCCCCATTCAGATCCTC
BZ-pUQ2-CysR-InvF2	GACATCCAGATGACCCAGTCTCCATC
BZ-UQ2A7-CysR-InsR	GGTCATCTGGATGTCCATATGATATCTCCTTCTAG
InfuEcoRISARS-CoV-2-A7VH back	CTTGCACTTGTCACGAATTCGGAGGTGCAGCTGTT GGAGT
InfuNheIXhoInCoV-2-A7VHfor	GATGGGCCCTTGGTGCTAGCGCTCGAGACGGTGA CCAGGGTT
InfuEcoRISARS-CoV-2-A7VLback	TCTTGCACTTGTCACGAATTCAGACATCCAGATGA CCCA
BsiWISARS-CoV-2-A7 VLfor	AGCCACCGTACGTTTGATTTCCACCTTGGTCCCTT
OverlapCKBack	AAATCAAACGTACGGTGGCTGCACCATCTGT
InfusionNheICKFor	TCATGTCGAGCTAGCTCCCTCTAACACTCTCCCCT
hACE2CDSFor	AGTGTGGTGGAATTCATGTCAAGCTCTTCCTGGCT CCTTC
hACE2CDSRev	CTTGCTCACCATCTCGAGCGGCCGCCACTGAAAGG AGGTCTGAACATCATCAG

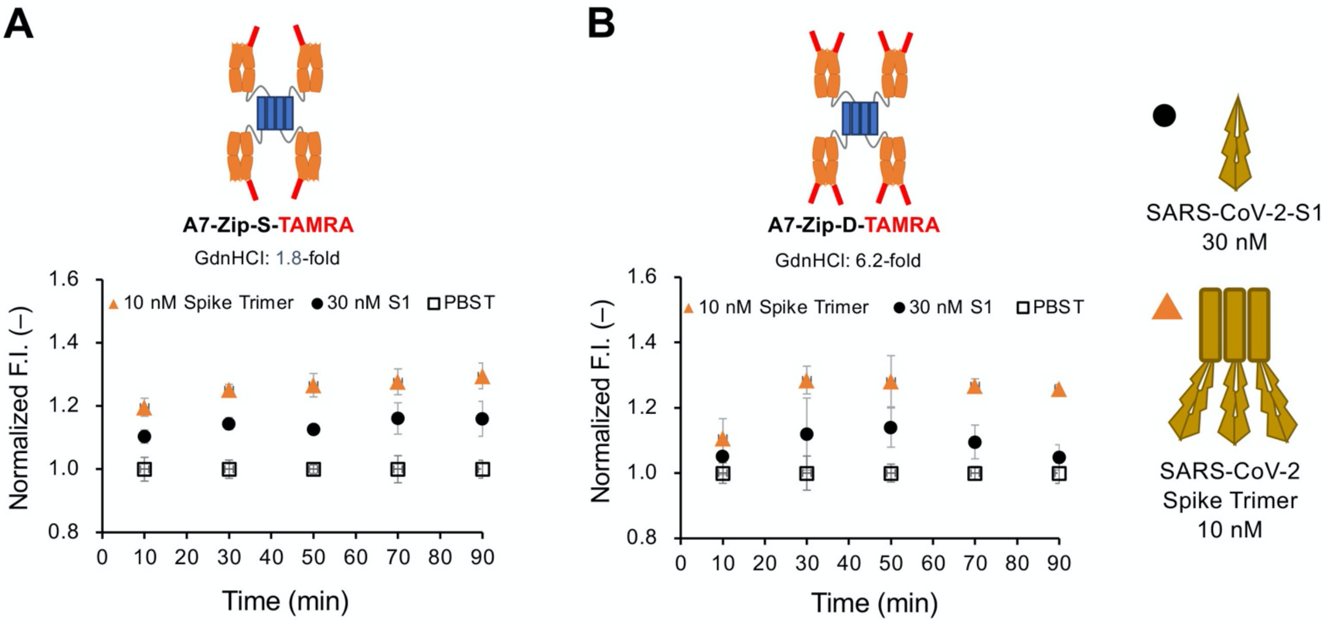

Supplementary Figure S1. Response of A7-Zip-S-TAMRA (A) and A7-Zip-D-TAMRA (B) Q-bodies to SARS-CoV2 spike protein S1 and full trimer over time. GdnHCl: Fluorescence intensity change of denatured Q-body; n = 4; Data are expressed as mean ± standard deviation.

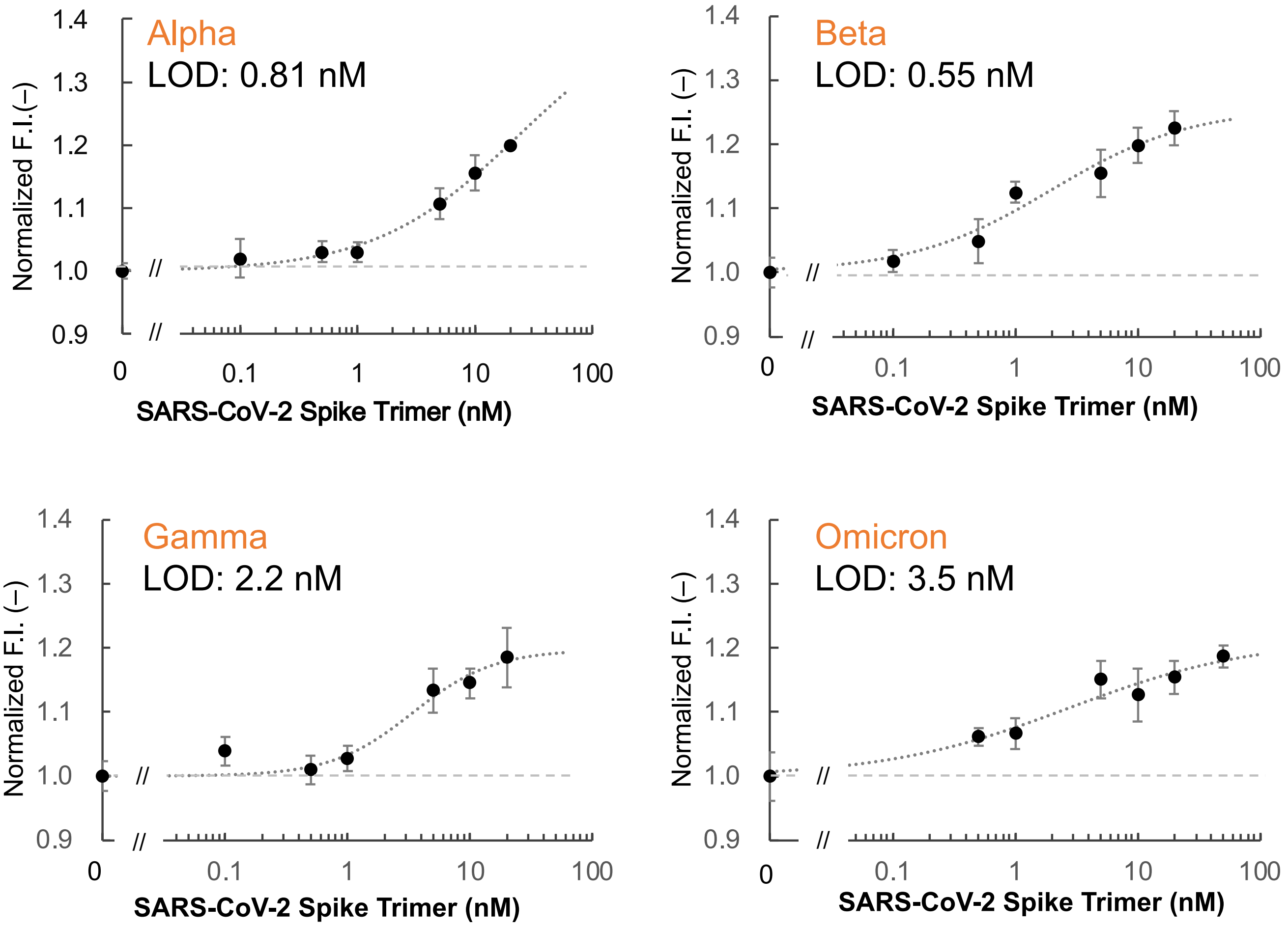

Supplementary Figure S2. Dose-response curves for SARS-CoV-2 spike protein variants with the A7-Zip-S-TAMRA Q-body. Buffer: PBST; n = 4; Data are expressed as mean ± standard deviation. Alpha: spike protein of SARS-CoV-2 variant alpha, 40589-V08B6, Sino Biological; Beta: spike protein of SARS-CoV-2 variant beta, 40589-V08B7, Sino Biological; Gamma: spike protein of SARS-CoV-2 variant gamma, 40589-V08B8, Sino Biological; Omicron: spike protein of SARS-CoV-2 variant of concern omicron, 40589-V08H26, Sino Biological.

A

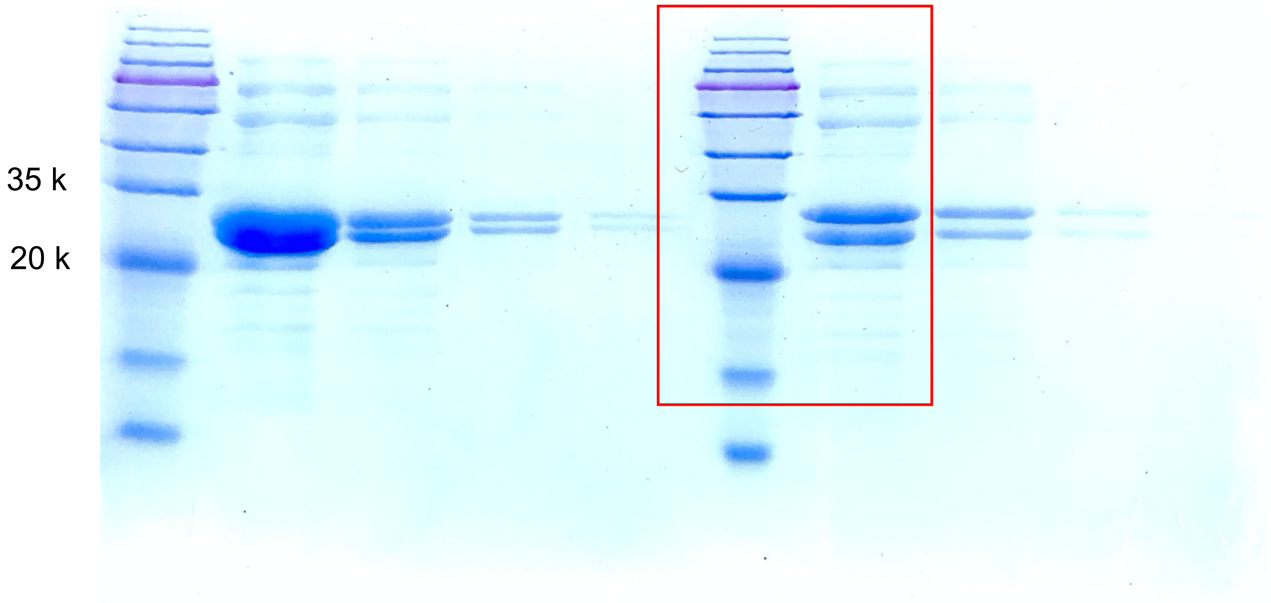

B

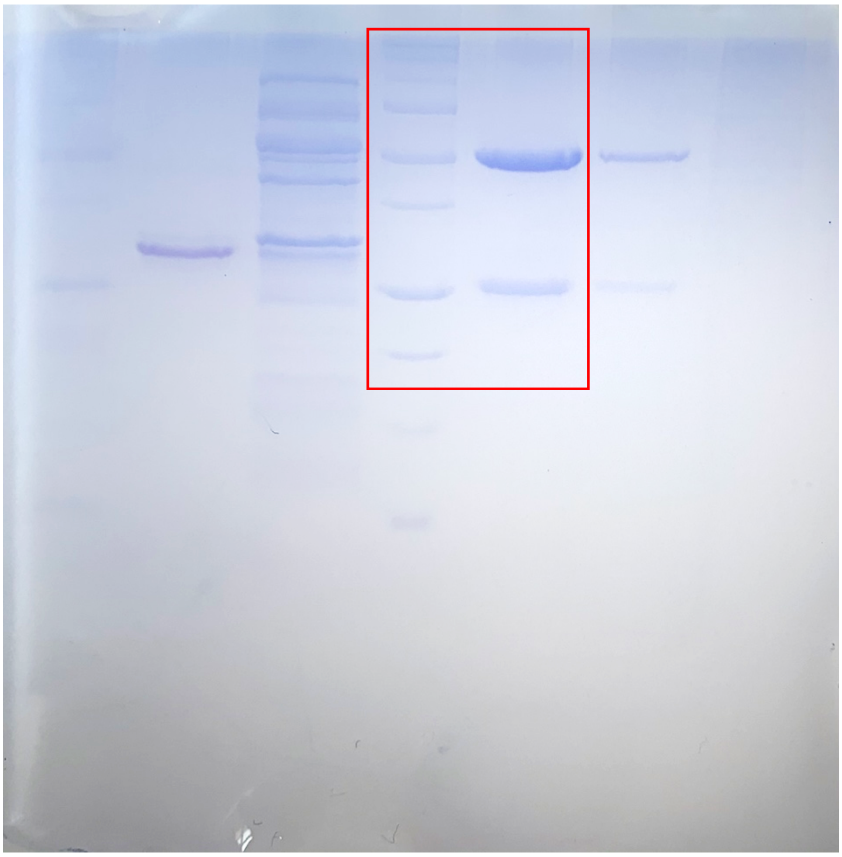

Supplementary Figure S3. Original SDS-PAGE pictures for those presented in main text. Those for Figure 2B (A) and Figure 5B (B) are shown.

A

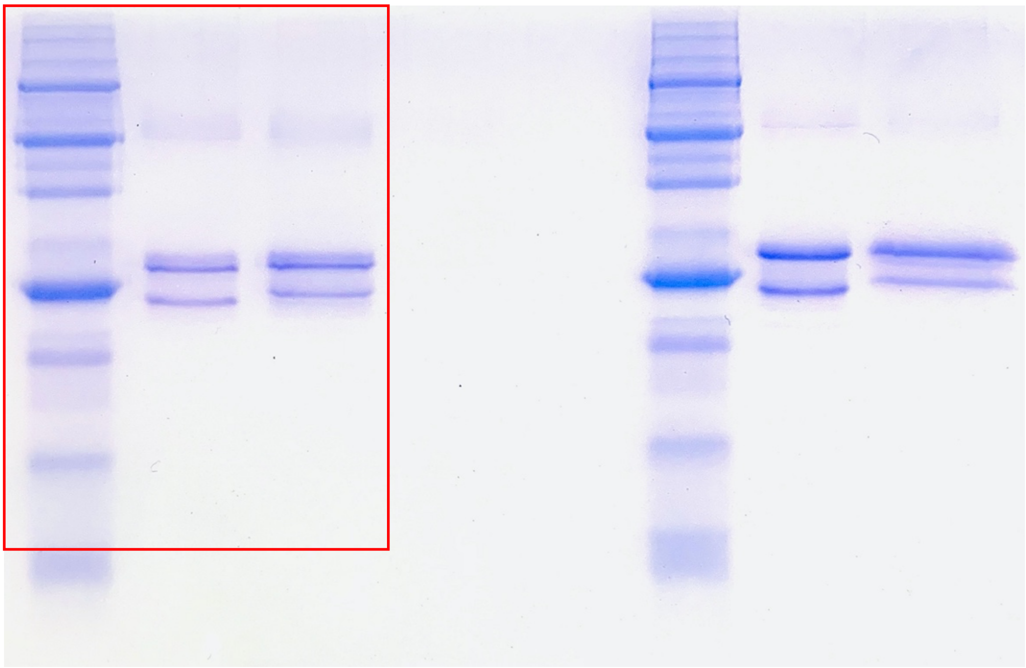

B

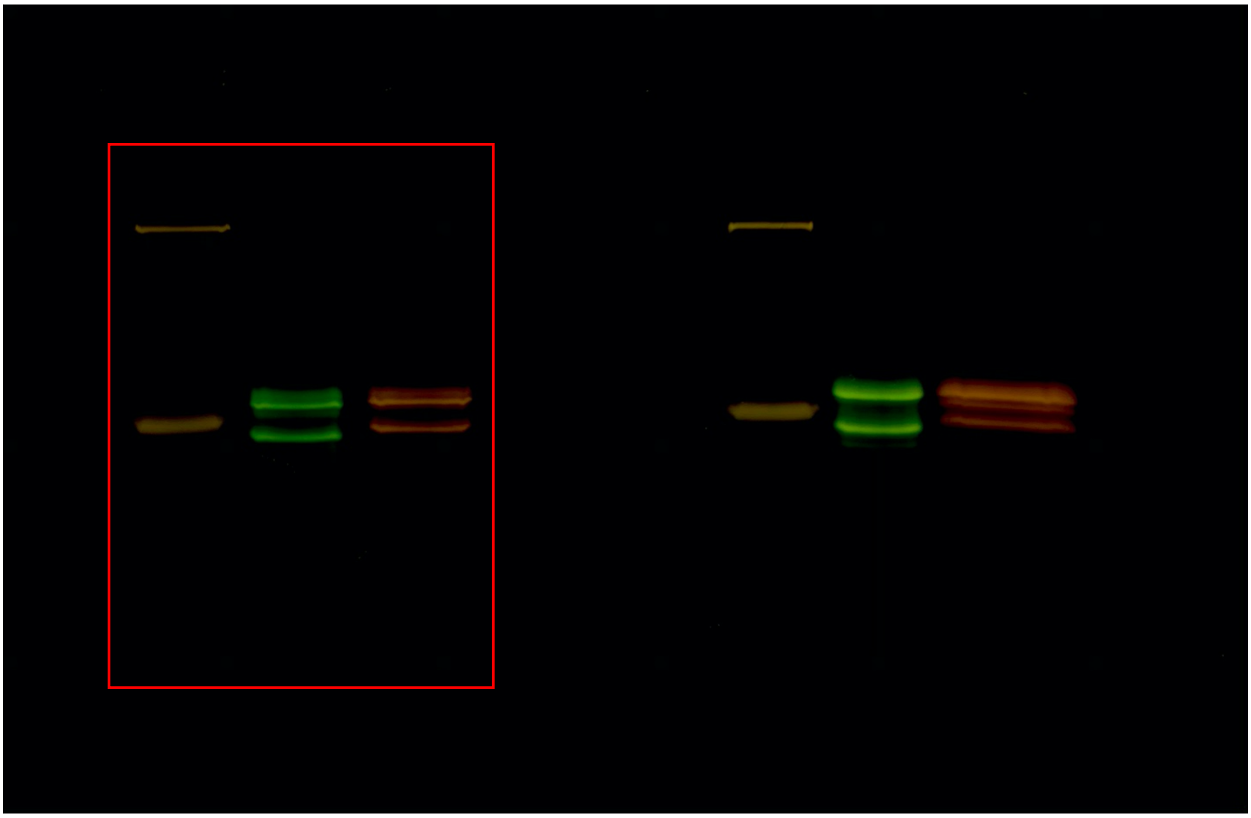

Supplementary Figure S4. Original gel pictures for Figure 7B. CBB-stained (A) and fluorescence image of the SDS-PAGE gel (B) are shown.
